# Practical Approaches to Identifying & Addressing Hearing Loss among Persons Living with Dementia

**DOI:** 10.1002/alz70857_101399

**Published:** 2025-12-25

**Authors:** Carrie L Nieman

**Affiliations:** ^1^ Cochlear Center for Hearing & Public Health, Johns Hopkins Bloomberg School of Public Health, Baltimore, MD, USA; Johns Hopkins University School of Medicine, Baltimore, MD, USA

## Abstract

Hearing loss is one of the most common comorbidities among persons living with dementia. Approximately 80% of persons living with dementia have a clinically significant hearing loss based on nationally representative US‐based cohorts, using audiometric data, increasing to >90% among patients who are 80 years and older. As age‐related conditions, the prevalence of hearing loss increases with age and is higher among those with greater severity of cognitive impairment. Proxy‐rated hearing loss by care partners and clinicians underestimate hearing loss among older adults with cognitive impairment. While often considered a benign part of the aging process, hearing loss among individuals with cognitive impairment is associated with increased number and severity of neuropsychiatric symptoms and hearing aid use may be protective. However, few persons living with dementia use hearing aids, with national‐representative estimates of around 20%. This presentation will review the current epidemiological understanding of hearing loss and its impact on persons living with dementia. The session will cover the clinical presentation of hearing loss, the basics of age‐related hearing loss, and practical tools for the assessment and management of hearing loss that can be integrated into routine clinical practice. We will cover tablet‐ and smartphone‐based screening options, over‐the‐counter hearing technologies, and best practices in counseling patients on hearing loss and its role in aging well with dementia.

